# Onion Peel Ethylacetate Fraction and Its Derived Constituent Quercetin 4′-*O*-β-D Glucopyranoside Attenuates Quorum Sensing Regulated Virulence and Biofilm Formation

**DOI:** 10.3389/fmicb.2017.01675

**Published:** 2017-09-05

**Authors:** Hanan M. Al-Yousef, Atallah F. Ahmed, Nasser A. Al-Shabib, Sameen Laeeq, Rais A. Khan, Md T. Rehman, Ali Alsalme, Mohamed F. Al-Ajmi, Mohammad S. Khan, Fohad M. Husain

**Affiliations:** ^1^Department of Pharmacognosy, College of Pharmacy, King Saud University Riyadh, Saudi Arabia; ^2^Department of Food Science and Nutrition, Faculty of Food and Agricultural Sciences, King Saud University Riyadh, Saudi Arabia; ^3^Department of Applied Chemistry, Faculty of Engineering and Technology, Aligarh Muslim University Aligarh, India; ^4^Department of Chemistry, College of Sciences, King Saud University Riyadh, Saudi Arabia; ^5^Department of Agricultural Microbiology, Aligarh Muslim University Aligarh, India

**Keywords:** antibiotic resistance, quorum sensing, biofilm formation, virulence factors, molecular docking

## Abstract

The resistance and pathogenesis of bacteria could be related to their ability to sense and respond to population density, termed quorum sensing (QS). Inhibition of the QS system is considered as a novel strategy for the development of antipathogenic agents, especially for combating drug-resistant bacterial infections. In the present study, the anti-QS activity of Onion peel ethylacetate fraction (ONE) was tested against *Chromobacterium violaceum* CV12472 and *Pseudomonas aeruginosa* PAO1. ONE inhibit the QS-mediated virulence factors production such as violacein in *C. violaceum* and elastase, pyocyanin in *P. aeruginosa*. Further, the treatment with sub-MICs of ONE significantly inhibited the QS-mediated biofilm formation, EPS (Extracellular polymeric substances) production and swarming motility. Further, quercetin 4′-*O*-β-D glucopyranoside (QGP) was isolated from ONE and its anti-QS potential was confirmed after observing significant inhibition of QS-controlled virulence factors such as violacein, elastase, pyocyanin and biofilm formation in test pathogens. Molecular docking analysis predicted that QGP should be able to bind at the active sites of Vfr and LasR, and if so blocks the entry of active sites in Vfr and LasR.

## Introduction

Development of multi-drug resistant bacteria has rendered the current antibiotic therapy more or less ineffective, and thus microbial infections have emerged as a major public health concern across the globe (Faheem et al., 2013; Khan and Rehman, 2016; Muteeb et al., 2016). To overcome this problem of drug resistance, the scientific community is looking for alternative strategies. One such novel drug target in bacteria is Quorum sensing (QS) which is density dependent cell-cell communication system that regulates the expression of an array of genes associated with the virulence of the pathogens (Fuqua et al., 2001). QS inhibitors do not impose selection pressure as they target processes that are important in the pathogenesis but are not essential for the survival of the pathogens (Rasmussen and Givskov, 2006).

Acyl-homoserine lactone (AHL) based QS systems are the most well studied and are known to regulate various functions in Gram-negative bacteria, e.g., bioluminescence, biofilm formation, sporulation, secondary metabolite production and virulence factor production (Camilli and Bassler, 2006; Reading and Sperandio, 2006). Therefore, AHL-regulated QS is often targeted using natural products to develop novel QS inhibitors. Various plants extracts (Adonizio et al., 2008; Musthafa et al., 2010; Zahin et al., 2010) and plant isolated secondry metabolites such as naringenin, kaemferol, quercetin, caffeine, menthol, and ajoene have demonstrated varying levels of QS and biofilm inhibition in Gram-negative pathogens (Vandeputte et al., 2011; Vasavi et al., 2014; Husain et al., 2015a,b; Vadekeetil et al., 2015). Quercetin, a major dietry flavonoid constiuent, ubiquitously present in plants including red onions, is shown to posses numerous biological activities including antibiofilm and anti-QS (Gopu et al., 2015; Tripoli et al., 2007). As a major component, quercetin and its derivatives isolated from phenolic rich fraction of different plants has shown significant antibiofilm/anti-QS activity against food borne as well as human pathogenic bacteria and could be held responsible for overall actvity of the extracts/fraction (Lee et al., 2013; Vasavi et al., 2016). However, the vast majority of plants and their isolated constituents phytochemicals are yet to be explored for their QS inhibitory properties.

Onion (*Allium cepa L*.) belonging to the Liliaceae family is one of the most economically important species (Sujitha et al., 2012). Besides its significant nutritional contribution to the human diet, onion is reported to have various medicinal properties, and it has been used as an herbal medicine for a long period of time (Bordia et al., 1975; Kawamoto et al., 2004; Rose et al., 2005). Antimicrobial property of the onion extract is also well explored against both Gram-negative and Gram-positive bacteria (Santas et al., 2010).

Considering the various medicinal and functional properties of onion, a study was planned with the aim to examine the QS and biofilm inhibitory activity of onion peel. Different fractions obtained by liquid liquid extraction method, employing series of steps (Jones and Kinghorn, 2005) were initially screened for anti-QS activity. The most active ethylacetate fraction (ONE) and its isolated constituent quercetin 4′-*O*-β-D glucopyranoside (QGP) were then evaluated for their anti-infective potentials against Gram-negative pathogenic bacteria. Molecular docking of the isolated compound (QGP) with different QS proteins were carried out to get enhanced mechanistic insight.

## Materials and Methods

### Plant Material

*Allium cepa* L, red onion, belonging to Granex hybrid cultivars was purchased from Hail farms, voucher number ATA32-1 was assigned to it and kept in Pharmacognosy Department, College of Pharmacy, King Saud University. The outer red skin was collected and then ground to coarse powder.

### Extraction and Isolation of QGP

The extraction was carried out according to the method Onion peel powder (1 kg) was extracted with 70% acetone in distilled water at room temperature for 48 h, till exhaustion. The extracts were combined and filtered. The filtrate was evaporated to dryness under reduced pressure, using the rotary vacuum at 45°C, to yield a dark brown gummy extract (156.2 g, yield 15.62% w/w). This extract was dissolved in 30% methanol in distilled water (0.5 L). Hydro-methanolic solution was successively fractionated with petrol ether (3 L × 0.5 L), chloroform (3 L × 0.5 L), ethyl acetate (5 L × 0.5 L), and *n*-butanol saturated with water (5 L × 0.5 L). Each fraction was concentrated under reduced pressure to give solvent-free residues: Onion peel petrol ether fraction (ONP) 1.2 g, onion chloroform fraction (ONC) 3.0 g, onion peel ethylacetate fraction (ONE) 80.0 g and onion peel *n-*butanol fraction (ONB) 41.0 g, respectively. The remaining aqueous layer was concentrated to yield onion peel aquous fraction (ONA) 29.0 g.

Twenty-five grams (out of total 80.0 g) of the ONE fraction was subjected to normal phase chromatography (Si gel 500 g, 1.5 m × 4 cm) packed by a wet method using CHCl_3._ MeOH in CHCl_3_ was used as an eluent in a gradient mode. The collected fractions (124 fractions) were then concentrated to dryness under reduced pressure at 40°C and then monitored by Si gel TLC using solvent systems CHCl_3_: MeOH: glacial AcOH, (8.7: 1.3: 0.2) which were then sprayed with Ce(SO_4_)_2_ to visualize the spots. Similar fractions were then pooled to give 15 sub-fractions. Fraction E8 eluted by 7% MeOH in CHCl_3_ deposited yellow solid that were purified by recrystallization to give a pure compound QGP (45 mg). The compound showed *R*_f_ = 0.46 (Si gel TLC, EtOAc : MeOH : H_2_O, 30:5:4).

### Characterization and Structure Elucidation of QGP

Quercetin 4′-*O*-β-D glucopyranoside gave a positive response (blue to green color) with 5% FeCl_3_ spray reagent on Si gel TLC and thus revealed their phenolic property. QGP is soluble in MeOH and when dissolved in dilute alkali gave an intense yellow solution. An intense yellow color was also obtainedon addition of 5% AlCl_3_ reagent which may indicate their flavonoid nature. QGP gave positive Molisch’s test after hydrolysis with H_2_SO_4_ designating its glycosidal nature. Ultraviolet absorption spectra were obtained using a Shimadzu UV-160/PC UV-Vis Spectrophotometer. The UV spectra of QGP in MeOH showed two absorption bands at 300, and 380 nm corresponding to cinnamoyl (Band I) and at 240–280 nm corresponding to benzoyl (Band II) moieties characteristic for flavonoids (Harborne et al., 1975). Other spectral analyses including MS and NMR spectroscopy were used for further structural elucidation of this compound.^1^H and ^13^C NMR spectra of the isolated compounds were recorded in deuterated dimethyl sulfoxide (DMSO-d6) on a Bruker AM500 instrument (Central Lab. at the College of Pharmacy, King Saud University, Bruker Biospin GmbH, Rheinstetten, Germany) operating at 500 MHz for protons and 125 MHz for carbons, respectively. The chemical shift values were reported in δ (ppm) units relative to the internal standard (TMS) and the coupling constants (J) were expressed in Hertz (Hz). Standard pulse sequences were used for generating COSY, HSQC, and HMBC spectra. The high resolution electron impact ionization-mass spectra (HREI-MS) were obtained on a solid probe using Shimadzu QP-2010 plus.

### Bacterial Strains and Growth Conditions

Bacterial strains used in this study were *Chromobacterium violaceum* CV12472, *Pseudomonas aeruginosa* PAO1 (McLean et al., 2004), and two clinical strains (*P. aeruginosa* PAF79 and *Aeromonas hydrophila* WAF38 isolated from diabetic foot infections). Bacteria from diabetic foot infection where isolated at Centre for Diabetes and Endocrinology, J.N.M.C, A.M.U. Aligarh, India using standard methods (Collee and Marr, 1996; Zubair et al., 2011). All the bacterial strains were grown in Luria-Bertani (LB) medium (Oxoid) at 30°C for 24 h.

### Determination of Minimum Inhibitory Concentration (MIC)

Minimum inhibitory concentrations of ONE and QGP were determined against selected pathogens using broth macro dilution method (Eloff, 1998; CLSI, 2004). Treated pathogens were incubated overnight (16–18 h) at 37°C in Mueller-Hinton broth and observed for turbidity. Least concentration at which no visible growth was observed was defined as the minimum inhibitory concentration. Concentrations below the MIC (Sub-MICs) were selected for the assessment of anti-virulence and anti-biofilm activity in the above test strains.

### Quantitative Estimation of Violacein

The extent of violacein production by *C. violaceum* CV12472 in the presence of sub-MICs of test agents was studied by extracting violacein and quantifying photometrically using the method of Blosser and Gray (2000) with little modifications (Husain et al., 2015a). One-ml culture from each flask was centrifuged at 16,000 ×*g* for 10 min to precipitate the insoluble violacein. The culture supernatant was discarded and 1 ml of DMSO was added to the pellet. The solution was vortexed vigorously for 30 s to completely solubilize violacein and centrifuged at 16,000 ×*g* for 10 min to remove the cells. Two hundred microliters of the violacein-containing supernatants were added to 96-well flat-bottomed microplates (Polylab, India), four wells per each solution and the absorbance was read with a microplate reader (Thermo Scientific Multiskan Ex) at a wavelength of 585 nm. Reduction in the production of pigment in the presence of test agents was measuredin terms of percent (%) inhibition as, [(OD of control – OD of treated)/OD of control] × 100.

### Effect on Virulence Factor Production

Effect of sub-MICs of test agents on virulence factors of *P. aeruginosa* such as LasB elastase, pyocyanin, swarming motility, EPS extraction and quantification was assessed as described previously (Husain and Ahmad, 2013).

### Assay for Biofilm Inhibition

The effect of test agents on biofilm formation was measured using the microtitre plate assay (O’Toole and Kolter, 1998). Briefly, 1% overnight cultures (0.4 OD at 600 nm) of test pathogens were added into1 mL of fresh LB medium in the presence and the absence of sub-MICs of test agents. Bacteria were allowed to adhere and grow without agitation for 24 h at 30°C. After incubation, microtitre plate was emptied by removing the media along with free-floating planktonic cells and the wells were gently rinsed twice with sterile water. The surface-attached cells (biofilm) were stained with 200 μL of 0.1% crystal violet (CV) (Hi-media, Mumbai, India) solution. After 15 min, CV solution was discarded completely, and wells were filled with 200 μL of 95% ethanol to solubilize CV from the stained cells. The biofilm biomass was then quantified by measuring the absorbance at OD 470 nm in a microplate reader (Thermo Scientific Multiskan Ex, India).

### Molecular Docking Analysis

The molecular docking analysis of the interaction between QGP and virulence factor (LasR and Vfr) was performed using Autodock 4.2 as described previously (Rehman et al., 2014; Husain et al., 2015a). The X-ray crystal structures of LasR (2UV0) and Vfr (3SZT) were downloaded from Protein Databank^1^. The PDB file of ligand (QGP) was prepared in the ChemDraw. Before performing molecular docking, a valid docking protocol was established by (i) extracting the natural ligand from the protein–ligand X-ray crystal structure PDB file (ii) re-docking the ligand with the protein, and (iii) comparing the docked protein–ligand complex with the X-ray structure of the complex.

The target proteins were processed by removing any heterogeneous compounds and water molecules. Further, polar hydrogen atoms and Kollman charges were added using Autodock tool. Affinity grid maps were generated in such a way as to cover the complete active sites of the respective proteins. Molecular docking was performed using Lamarkian Genetic Algorithm (LGA) to calculate the possible conformations of the ligand that binds to the target proteins. Here, the ligand was set free to search and bind at the active site of the protein in the most favorable or minimum energy conformation. Initial positions and orientations of the ligand were set randomly while the torsions were set to a maximum of 6. Each run of the docking was performed to calculate 2500000 energy evaluations. The population size, translational step, quaternion and torsion steps were set to 150, 0.2 and 5, respectively. On the basis of binding energy (ΔG), best docked structures were saved and analyzed for receptor–ligand interactions using Discovery Studio 4.0 (Accelrys Software Inc., 2012). The binding constant (*K*_b_) for protein–ligand interaction was calculated using the following relation (Rehman et al., 2015):

$$\Delta G=-RT\;\ln\;K_{b}$$

where, *R* is the gas constant (1.987 cal/mol/K) and *T* is the temperature (298 K).

### Statistical Analysis

All experiments were performed in triplicates and the data obtained from experiments were presented as mean values and the difference between control and test were analyzed using student’s *t*-test.

## Results and Discussion

### Compound QGP

The most active ethylacetate fraction (ONE) and its presumed constituent (QGP) was further screened to confirm its identity. A solution of QGP in MeOH gave a positive response (blue to green color) with 5% FeCl3 spray reagent on Si gel TLC, revealing its phenolic property. It is dissolved in dilute alkali to give an intense yellow solution. An intense yellow color was also obtained on addition of 5% AlCl3 reagent which indicated its flavonoid nature. Moreover, QGP gave positive Molisch’s test after hydrolysis with 0.1 N H_2_SO_4_ designating its glycosidal form. The UV spectra of QGP in MeOH showed two characteristic absorption bands at 300 and 380 nm corresponding to cinnamoyl (Band I) and at 240–280 nm corresponding to benzoyl (Band II) moieties characteristic for flavonoids (Harborne et al., 1975). Other spectral analyses including MS and NMR spectroscopy were used for structural elucidation of this compound. Compound QGP (Supplementary Figure S1) was isolated as a yellow powder, m.p. = 209–210°C, *Rf* 0.64 (Si gel TLC, EtOAc- MeOH- H2O, 30: 5: 0.25). The compound (in MeOH) exhibited typical UV absorption spectrum of flavonoids. The ^13^C NMR spectrum of QGP, measured in DMSO-d6, displayed 21 carbon signals (Supplementary Table S1). The molecular ion peak appearing in the ESI-MS (-ve) at *m/z* 463 (Supplementary Figure S2) together with the NMR data concluded the molecular formula of QGP to be C_21_O_12_H_20_ of a flavonoid monoglycoside. Six *sp^3^* carbon signals of five oxymethine and one oxymethylene groups were found to be HSQC correlated with the proton signals at δH3.21 – 4.86 of a glucose moiety. Furthermore, comparison of NMR data of the aglycone moiety resulted from acid hydrolysis of QGP with the published data revealed that QGP is a quercetin glucoside. The 4′ location of sugar moiety was established on the basis of the significant downfield shift observed for the quaternary carbon C-1′ (ΔδC = 3.2 ppm) and H-5′ (ΔδC = 0.39 ppm) relative to those of aglycone. Furthermore, the 4′-linkage of glucose was confirmed by the HMBC correlation that observed from the sugar anomeric proton H-1″ (δH4.86) to C-4′ of ring B (δC146.7) of the aglycone moiety, (Supplementary Figure S3). From the above findings, QGP was identified as quercetin 4′-*O-*β-D-glucopyranoside (spiraeoside) and was further confirmed by comparing NMR data with those reported previously (Fossen et al., 1998; Moco et al., 2006). The above discussed NMR data of QGP compound confirmed the presence of quercetin-4′-*O*-β-D-glucoside (spiraeoside) (Supplementary Figures S6–S11). This compound was previously reported in the inner pigmented scale leaves of the red onion bulb (Fossen et al., 1998).

### Determination of MIC

Minimum inhibitory concentration was determined for the extract of onion peel against the test *C. violaceum* CV12472 and *P. aeruginosa* PAO1. The extract inhibited growth against both pathogens, the MIC was found to be 500 μg/ml for *C. violaceum* CV12472 and *A. hydrophila* WAF38, 800 μg/ml for *P. aeruginosa* PAO1 and, 1200 μg/ml for the clinical strain (*P. aeruginosa* PAF79). Hence, in the present study, sub-MIC concentrations (50–600 μg/ml) of ONE were used for further assays.

### Growth Curve Assay

Bacterial growth curve assay was performed at sub-MICs of ONE against both bacterial pathogens, to confirm the non-antibacterial activity of the extract. Growth curve analysis revealed no significant difference in the cell densities of the test pathogen in broth with or without 400 μg/ml ONE (Supplementary Figures S4A,B).

### Violacein Inhibition Assay

**FIGURE 1:**
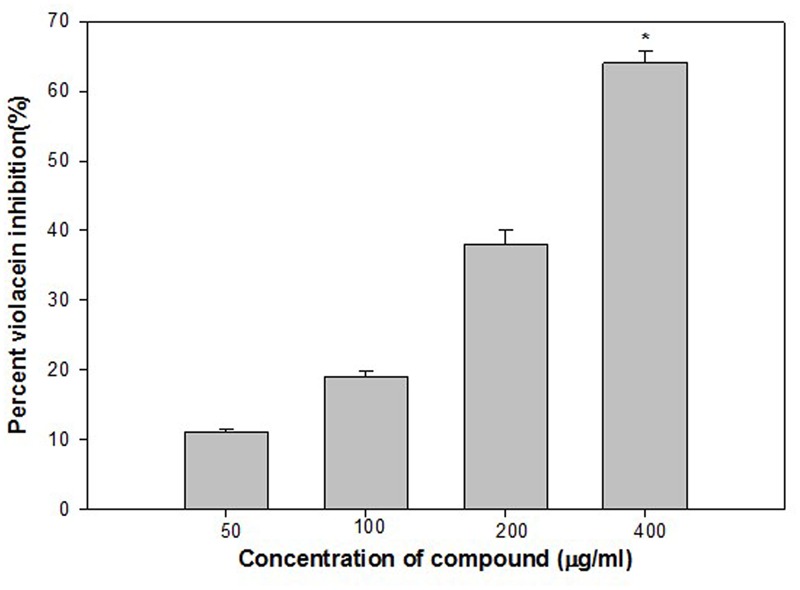
Quantitative assessment of violacein inhibition in CV12472 at sub-inhibitory concentrations of ethylacetate fraction of Onion peel (ONE) extract. All of the data are presented as mean ± standard deviation. ^∗^*p* ≤ 0.05, ^∗∗∗^*p* ≤ 0.001.

Violacein (purple pigment) production in *C. violaceum* is a QS regulated process, and its production is coordinated by CviIR-dependent QS system. In the present investigation, ONE inhibited violacein production in wild-type *C. violaceum* 12472 strain in a dose-dependent manner without affecting the growth of the bacteria. Maximum reduction of 64% was recorded at 400 μg/ml while at lower concentrations (50, 100, and 200 μg/ml) 11–38% decrease in violacein was observed (**Figure 1**). This concentration dependent action of ONE on violacein production is in accordance with the reports on Indian medicinal plants (Zahin et al., 2010), *Capparis spinosa* (Sybiya Vasantha Packiavathy et al., 2012), and *Cuminum cyminum* extract (Sybiya Vasantha Packiavathy et al., 2012).

### Effect of ONE on Virulence Factors of *P. aeruginosa* PAO1

**FIGURE 2:**
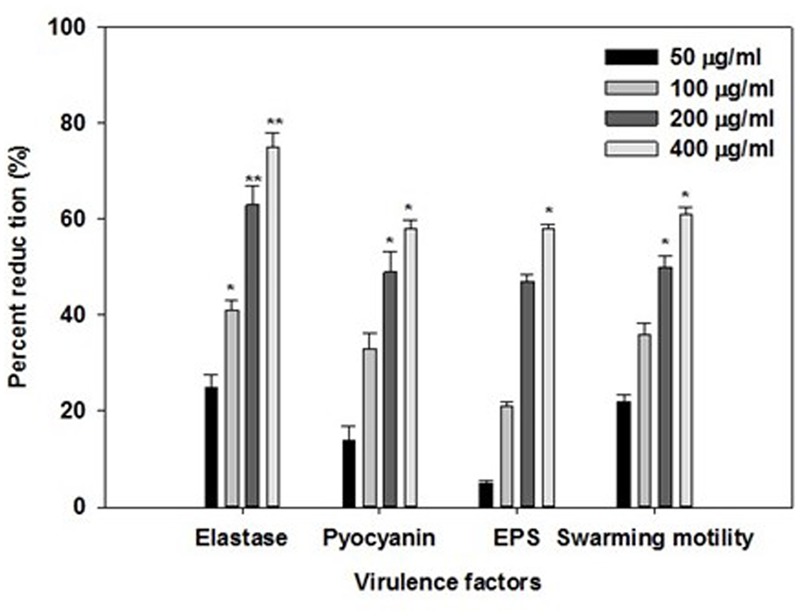
Effect of sub-MICs of ONE on quorum sensing regulated virulence factors in *P. aeruginosa* PAO1. The data represents mean values of three independent experiments. ^∗^*p* ≤ 0.05, ^∗∗^*p* ≤ 0.005.

Opportunistic human pathogen *P. aeruginosa* integrates AHL-dependent signaling with 4-quinolone dependent QS (Diggle et al., 2006). Therefore, the *las, rhl*, and *pqs* quorum-sensing systems of *P. aeruginosa* regulate the production of several extracellular virulence factors like elastase, the LasA protease, alkaline protease, motility, exopolysaccharide and pyocyanin (Latifi et al., 1995; Winson et al., 1998; de Kievit and Iglewski, 2000; Williams, 2007). Effect of sub-inhibitory concentrations of ONE on virulence factors of *P. aeruginosa* PAO1 is depicted in **Figure 2**. Statistically significant decrease in LasB elastase activity was observed in the culture supernatant of PAO1 treated with sub-MICs of ONE. A minimum of 25% inhibition was observed when PAO1 was cultured with ONE at a concentration of 50 μg/ml and maximum of 75% inhibition was observed at 400 μg/ml concentration of the extract. Elastase enzyme enhances the growth and invasiveness of the pathogen by degrading the structural components of the infected tissue (Kharazmi, 1989). In this present investigation, the ONE demonstrated concentration-dependent inhibition of elastase in PAO1, as shown in **Figure 2**. This result is in agreement with the study of Musthafa et al. (2010), who demonstrated significant inhibition of LasB activity by edible plants and fruits. Previous reports suggest that flavonid rich extracts of medicinal and edible plants exerts appreciable inhibitory effect against QS dependent expression of proteolytic enzymes such as LasB in PA01.

In addition to this recently, flavanones (Vandeputte et al., 2011), *Sclerocarya birrea* bark extract (Sarkar et al., 2014) and *Trigonella foenum-graceum* seed extract (Husain et al., 2015a) have been shown to inhibit elastase activity to substantial levels.

Production of blue colored pyocyanin is regulated by QS (Williams, 2007). Pyocyanin and its precursor phenazine-1-carboxylic acid (PCA) cause neutrophil apoptosis and impairs neutrophil-mediated host defenses (Fothergill et al., 2007). ONE at sub-lethal concentrations exhibited considerable decrease in the pyocyanin production by PAO1. The maximum reduction of 58% in pyocyanin production was recorded at highest tested concentration (400 μg/ml) followed by 49, 33, and 14% at 200, 100, and 50 μg/ml concentration, respectively (**Figure 2**). Our results are in accordance with the results of recent reports wherein Krishnan et al. (2012), and Gala et al. (2016) demonstrated that extracts of *S. aromaticum* (bud) and *Tinospora cordifolia* (stem) reduced the production of pyocyanin significantly.

Swarming motility and exopolysaccharide production by *P. aeruginosa* plays a vital role in the initiation, maturation, and maintenance of the biofilm architecture (Pratt and Kolter, 1998; Hentzer et al., 2003). Therefore, any interference with the motility and exopolysaccharide production is bound to affect the biofilm formation by the pathogen. In the present study, treatment of PAO1 with sub-MICs of ONE showed significantly reduced exopolysaccharide production, the extract (50–400 μg/ml) demonstrated inhibition in exoploysaacharide production to the level of 5–58%. Similarly, swarming migration of PAO1 was also impaired considerably (22–61%) after treatment with test concentrations of ONE (**Figure 2**). This statistically significant reduction of motility and exopolymeric material is previously reported with *Trigonella foenum-graceum* seed extract (Husain et al., 2015a).

**FIGURE 3:**
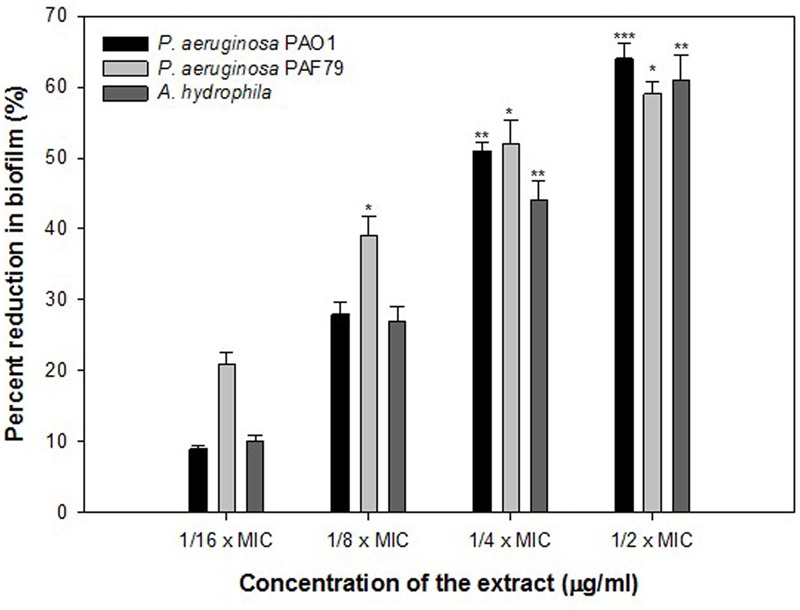
Quantitative measurement of biofilm inhibition as quantified by crystal violet staining and measuring absorbance at 470 nm. The data represents mean values of three independent experiments. ^∗^*p* ≤ 0.05, ^∗∗^*p* ≤ 0.005, ^∗∗∗^*p* ≤ 0.001.

Biofilm is a drug resistant complex aggregation of microorganisms and is a key factor in the pathogenesis of *P. aeruginosa* (Caraher et al., 2007). In a biofilm adherent cells become embedded within a slimy extracellular matrix that is composed of extracellular polymeric substances (EPS). Biofilms are the cause of severe persistent infection and biofilm formaton is considered as one of the potential drug targets to combat drug-resistant chronic infections (Hall-Stoodley et al., 2004; Wu et al., 2015). The ONE showed 9, 28, 51, and 64% decrease in the biofilm forming ability of PAO1 at 50, 100, 200, and 400 μg/ml of extract concentration, respectively (**Figure 3**). To explore the broad-spectrum biofilm inhibitory potential of ONE, we tested its sub-MICs against clinical strains (*P. aeruginosa* PAF79 and *A. hydrophila* WAF38). In PAF79 21–59% reduction in biofilm biomass was recorded while in *A. hydrophila* WAF38 10–61% decrease in biofilm was observed in comparison to the untreated control (**Figure 3**). Our observations find support from previous investigation on biofilm inhibition in PAO1 by polyphenolic extract of South Florida plants (Adonizio et al., 2008), *Lagerstroemia speciosa* fruit extract (Singh et al., 2012), *Rosa rugosa* (Zhang et al., 2014), standardized extract *of Sclerocarya birrea* (Sarkar et al., 2014), *Trigonella foenum-graceum* seed extract (Husain et al., 2015a) and *Mangifera indica* leaf extract (Husain et al., 2017).

### Molecular Docking Analysis

**FIGURE 4:**
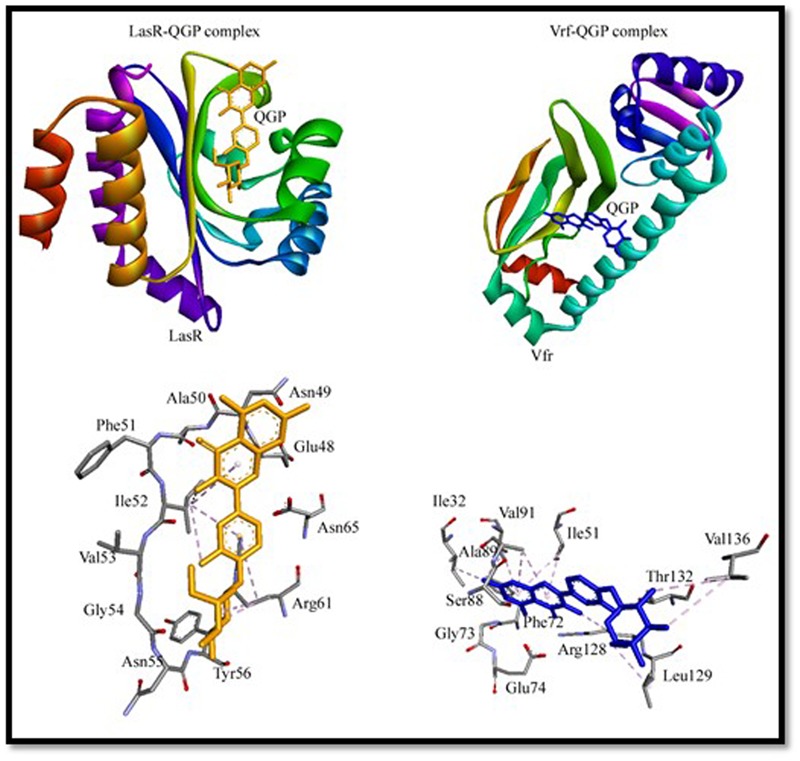
Molecular docking of quercetin 4′-*O*-β-D glucopyranoside (QGP) with LasR and Vfr.

To gain an insight into the binding mechanism of QGP to virulence factors (LasR and Vfr), we performed molecular docking using Autodock 4.2 and the results are presented in **Figure 4** and Supplementary Table S2. To validate the docking procedure, we first extracted natural ligands from their protein–ligand crystal structure and then again re-docked using Autodock 4.2. Supplementary Figure S5 shows a comparison between crystal structures and docked conformations of ligand–protein complexes. It can be seen from Supplementary Figure S5 that ligands occupied similar positions in the docked conformations as it was present in the crystal structures. We ascertain that the molecular docking procedures adopted in this study were valid.

The X-ray crystal structure of Vfr (3SZT) represents a complex between Vfr and cAMP bound at the active site (Cordes et al., 2011). The re-docking of cAMP into the active site of Vfr predicted that it was boundat the active site (Supplementary Figure S5 and Table S2). We found that cAMP interacted hydrophobically (π-alkyl interaction) with Ile51, Ile63, Ala89, and Arg128 of Vfr (Supplementary Figure S5 and Table S2). Docking of QGP to Vfr indicated that it was bound at the active site mainly through hydrophobic interactions. QGP interacted strongly with Vfr by forming 12 hydrophobic interactions with Ile32, Ile51, Arg87, Ala89, Val91, Arg128, Leu129, and Val136 (**Figure 4** and Supplementary Table S2). It should be noted that the residues Ile51, Ala89, and Arg128 of Vfr were commonly occupied by its natural ligand cAMP as well as QGP. Molecular docking study revealed that QGP-Vfr complex was stabilized by an estimated free energy of -6.83 kcal/mol compared to -8.91 kcal/mol of free energy in case of Vfr-cAMP interaction. The binding affinity of the Vfr-QGP complex was predicted to be the order of 10^5^ M^-1^ as compared to 10^6^ M^-1^ for Vfr-cAMP complex (**Figure 4** and Supplementary Table S2).

The X-ray crystal structure LasR (2UV0) is a complex of LasR and N-3-oxo-dodecanoyl-L-homoserine lactone or 3-oxo-C12-HSL (Bottomley et al., 2007). The re-docking of 3-oxo-C12-HSL to LasR predicted that it interacted with the active site residues of LasR (Supplementary Figure S5 and Table S2). The docking of QGP with LasR has given clues that it did not bind at the active site of LasR, however, we found that it blocked the access of the active site by binding at the entrance of the cavity (**Figure 4**). The residues involved in LasR-QGP interaction were Ile52 and Arg61. Other residues that surrounded QGP were Glu48, Asn49, Ala50, Phe51, Val53, Glu54, Asn55, Tyr56, and Asn65. It has been foreseen that QGP formed one electrostatic interaction and one hydrogen bond with Arg61. Overall, the molecular docking study expected that LasR-QGP complex was stabilized by seven hydrophobic interactions (**Figure 4** and Supplementary Table S2). The Gibb’s free energy of LasR-QGP interaction was predicted to be -5.98 kcal/mol, which correspond to a binding constant of the order of 10^4^ M^-1^. On the basis of molecular docking study, we found that interaction of QGP with LasR was weaker as compared to the binding of its natural ligand 3-oxo-C12-HSL. The Gibb’s free energy and binding constant for LasR-3-oxo-C12-HSL interaction has been estimated to be -9.10 kcal/mol and 10^6^ M^-1^, respectively.

It is envisaged that QGP binds to Vfr more strongly and favorably than LasR. The possible mode of action of QGP is thus believed to act by inhibiting the function of Vfr. Vfr is a key player in regulating QS mechanism. It is a member of winged-helix family of transcription regulators, which controls the transcription of lasR and Type III secretion system. It also regulates flagellar gene expression and mobility (Cordes et al., 2011).

### Evaluation of Quorum Sensing Inhibitory Activity of Quercetin 4′-*O*-β-D Glucopyranoside (QGP)

**FIGURE 5:**
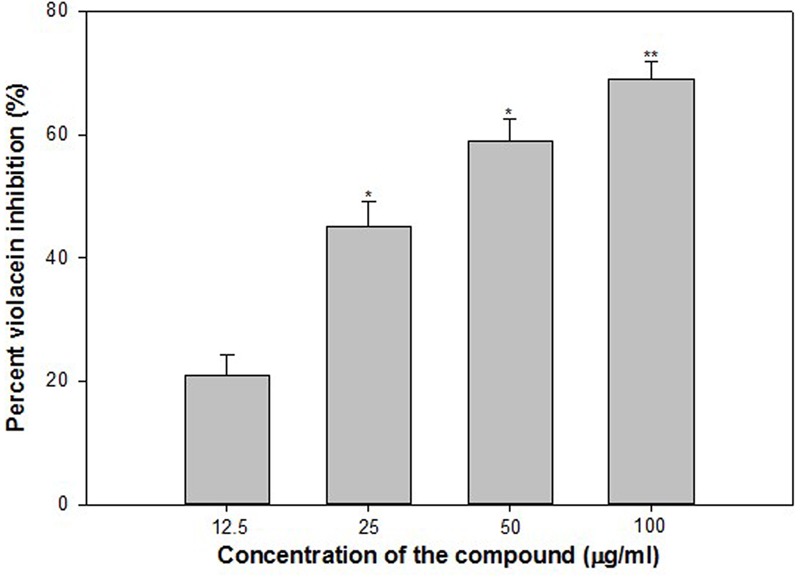
Quantitative assessment of violacein inhibition in CV12472 at sub-inhibitory concentrations of quercetin 4′-*O*-β-D glucopyranoside (QGP). All of the data are presented as mean ± standard deviation. ^∗^*p* ≤ 0.05, ^∗∗^*p* ≤ 0.005.

**FIGURE 6:**
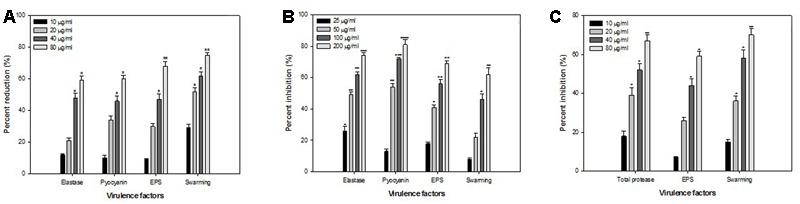
Effect of sub-MICs of QGP on quorum sensing regulated virulence factors. **(A)** *P. aeruginosa* PAO1; **(B)** *P. aeruginosa* PAF79; and **(C)** *A. hydrophila* WAF38. The data represents mean values of three independent experiments. ^∗^*p* ≤ 0.05, ^∗∗^*p* ≤ 0.005, ^∗∗∗^*p* ≤ 0.001.

**FIGURE 7:**
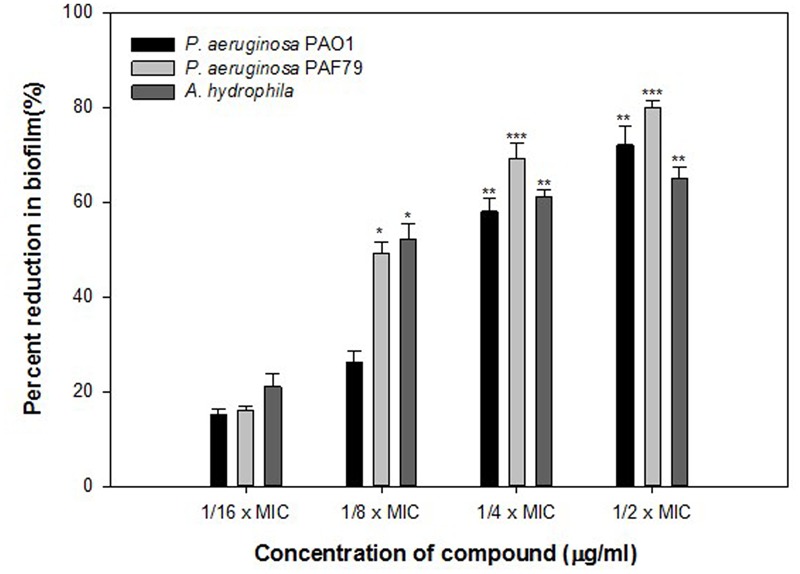
Quantitative measurement of biofilm inhibition in selected pathogenic bacteria as quantified by crystal violet staining and measuring absorbance at 470 nm. The data represents mean values of three independent experiments. ^∗^*p* ≤ 0.05, ^∗∗^*p* ≤ 0.005, ^∗∗∗^*p* ≤ 0.001.

Findings of the molecular docking with QGP were confirmed *in vitro* using the *C. violaceum* 12472 and *P. aeruginosa* PAO1 test strains. MIC of QGP was found to be 200, 400, and 100 against *C. violaceum* 12472, *P. aeruginosa* PAF79, *A. hydrophila* WAF38, and *P. aeruginosa* PAO1, respectively. At the tested sub-MICs (12.5–100 μg/ml) QGP demonstrated statistically significant inhibition of violacein pigment ranging from 21 to 69% over untreated control (**Figure 5**). IC_50_ value was found to be 30.98 μg/ml. QGP was further assessed for its anti-virulence properties in PAO1 and dose-dependent reduction in all the studied virulence factors was observed. Test compound (QGP) decreased the elastase activity (12–59%), pyocyanin production (10-60%), exopolysaccharide production (9–68%) and swarming motility (29–75%) at sub-inhibitory concentrations ranging from 10 to 80 μg/ml (**Figure 6A**). Effect of QGP on QS regulated virulence was also studied in two clinical strains, i.e., *P. aeruginosa* PAF79, *A. hydrophila* WAF38. At sub-MICs ranging from 25 to 200 μg/ml, 26–74% reduction in elastase activity, 13–81% decrease in pyocyanin production, 18–69% reduced EPS production and 8–62% impairment of swarming migration was recorded (**Figure 6B**). Further, in *A. hydrophila*WAF38 statistically significant reduction in total protease (18–67%), EPS production (7–59%) and swarming motility (15–70%) was observed at tested sub-MICs over untreated control (**Figure 6C**). Biofilm formation by PAO1 was also impaired by 15, 26, 58, and 72% at 10, 20, 40, and 80 μg/ml concentrations, respectively (**Figure 7**). Further, QGP significantly impaired the biofilm forming capabilities of *P. aeruginosa* PAF79 and *A. hyrophila* at respective sub-MICs. *P. aeruginosa* PAF79 biofilm was reduced by 16–80% at concentrations ranging from 25 to 200 μg/ml (**Figure 7**). While, 21–65% decrease in biofilm biomass of *A. hyrophila* after treatment with sub-inhibitory concentrations of QGP (**Figure 7**). In a similar study, quercetin without impacting the growth of PAO1, significantly inhibited (*P* < 0.05) biofilm formation and production of virulence factors including pyocyanin, protease and elastase at sub-lethal doses (Ouyang et al., 2016). Further, our findings are in accordance with other results published on flavanones (i.e., naringenin, eriodictyol, and taxifolin) (Vandeputte et al., 2011), methyl eugenol (Sybiya Vasantha Packiavathy et al., 2012), eugenol (Zhou et al., 2013) caffeine (Husain et al., 2015a), and menthol (Husain et al., 2015b).

## Conclusion

Onion is well known for its medicinal use and this study appends an additional note on its QS and biofilm inhibitory properties against pathogenic bacteria. The present study demonstrates that ONE could inhibit the QS mediated virulence factors production in *C. violaceum, P. aeruginosa*, and *A. hyrophila*. Further, the treatment with sub-MICs of ONE significantly inhibited the QS-mediated biofilm formation, EPS production and swarming motility in these pathogens. Further, QGP was isolated from the ethyl acetate fraction and was studied for anti-QS properties both *in silico* and *in vitro*. Broad-spectrum *in vitro* inhibition of QS-controlled virulence factors such as violacein, elastase, pyocyanin, EPS and biofilm in test pathogens was observed. Thus, these results suggest that ONP and its bioactive compound QGP may have potential anti-infective properties and could prove to be an effective anti-QS and antibiofilm agent against pathogens.

## Author Contributions

HA-Y, FH, AFA, and RK designed and conceived experiments. FH, HA-Y, SL, RK, NA-S, AA, and MR performed experiments. FH, HA-Y, SL, RK, AFA, and MA-A analyzed and interpreted the data. NA-S, FH, SL, HA-Y, RK, MR, MA-A, and MK wrote the manuscript and all authors reviewed it.

## Conflict of Interest Statement

The authors declare that the research was conducted in the absence of any commercial or financial relationships that could be construed as a potential conflict of interest.

## Footnotes

1 http://www.rcsb.org/pdb/
