# Supplementary material for: Onion Peel Ethylacetate Fraction and Its Derived Constituent Quercetin 4′-O-β-D Glucopyranoside Attenuates Quorum Sensing Regulated Virulence and Biofilm Formation
Source: Front Microbiol. 2017 Sep 5;8:1675. doi: 10.3389/fmicb.2017.01675 (PMC5591837; doi:10.3389/fmicb.2017.01675)
Supplement: Supplementary file 1 [file Data_Sheet_1.docx]

**Supplementary material**

**Onion Peel Ethyl acetate Fraction and Its Derived Constituent Quercetin 4`-O-*Β*-D Glucopyranoside Attenuates Quorum Sensing Regulated Virulence and Biofilm Formation**

Hanan M. Al-Yousef^1*^, Atallah F Ahmed^1^, Nasser A. Al-Shabib^2^, Sameen Laeeq^3^, Rais Ahmad Khan^4^, Md Tabish Rehman^1^, Ali Alsalme^4^, Mohamed F Al-Ajmi^1^, Mohammad Shavez Khan^5^, Fohad Mabood Husain^2*^

****Corresponding Authors***

**Dr. Fohad Mabood Husain**

Department of Food Science and Nutrition

College of Agriculture and Food Science

King Saud University, Riyadh-11451, KSA

fahadamu@gmail.com, fhussain@ksu.edu.sa

**Dr. Hanan M. Al-Yousef**

Department of Pharmacognosy,

College of Pharmacy,

King Saud University, Riyadh, Saudi Arabia

halyousef@ksu.edu.sa

**
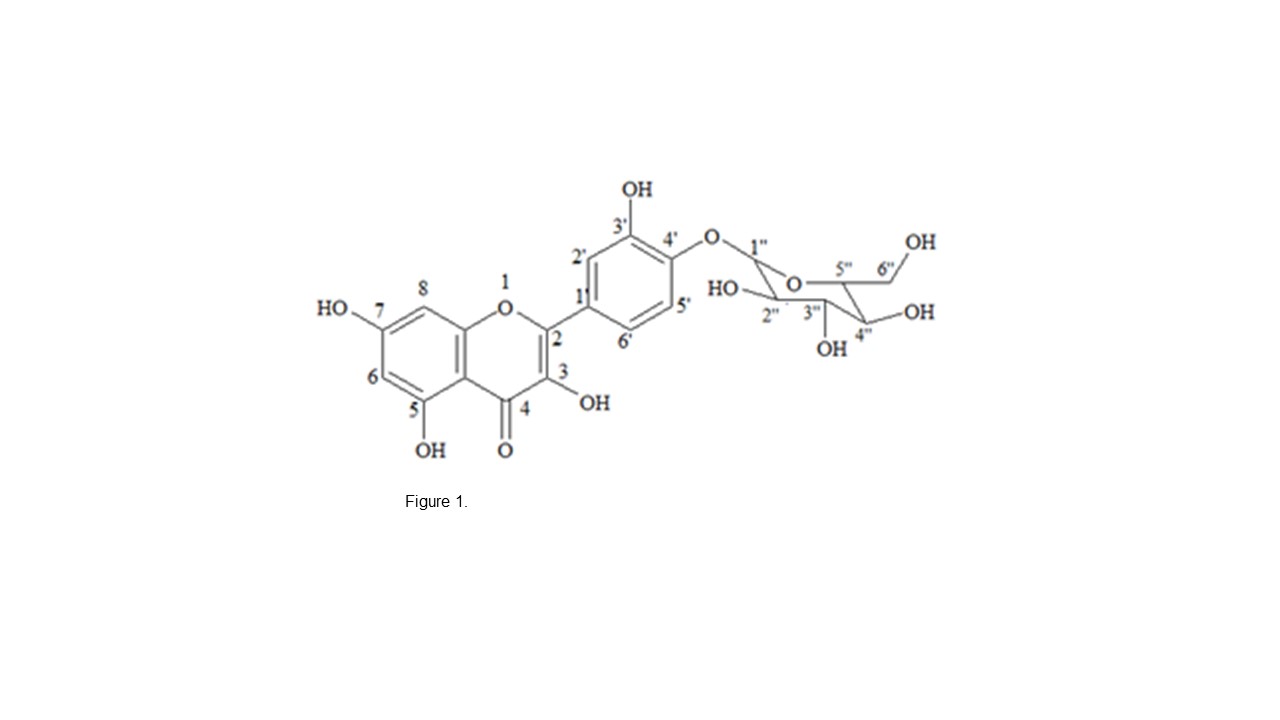
**

**Figure S1.** Structure of quercetin 4`-O-β-D glucopyranoside (QGP**)**

**
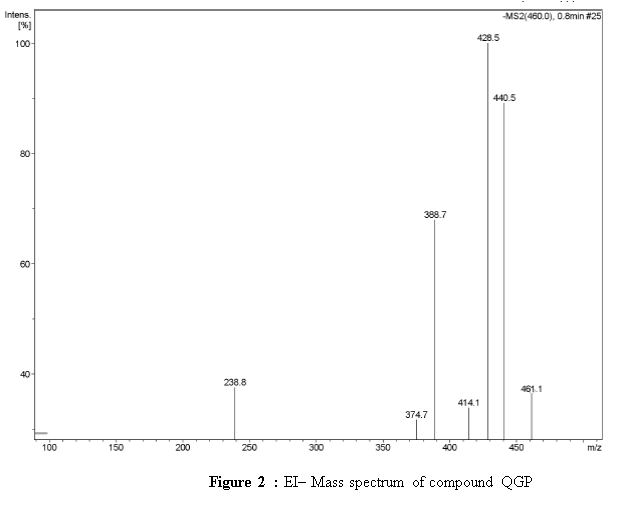
**

**Figure S2.** ESI-Mass spectrum of QGP

**
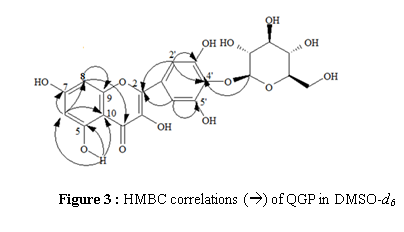
**

**Figure S3**. HMBC correlations (🡪) of QGP in DMSO-*d_6_*

A

**Figure S4:** Growth characteristic of *P. aeruginosa* PAO1 (A) and *A. hydrophila* WAF38 (B) in Luria–Bertani (LB) medium in the absence and presence of ONE

**
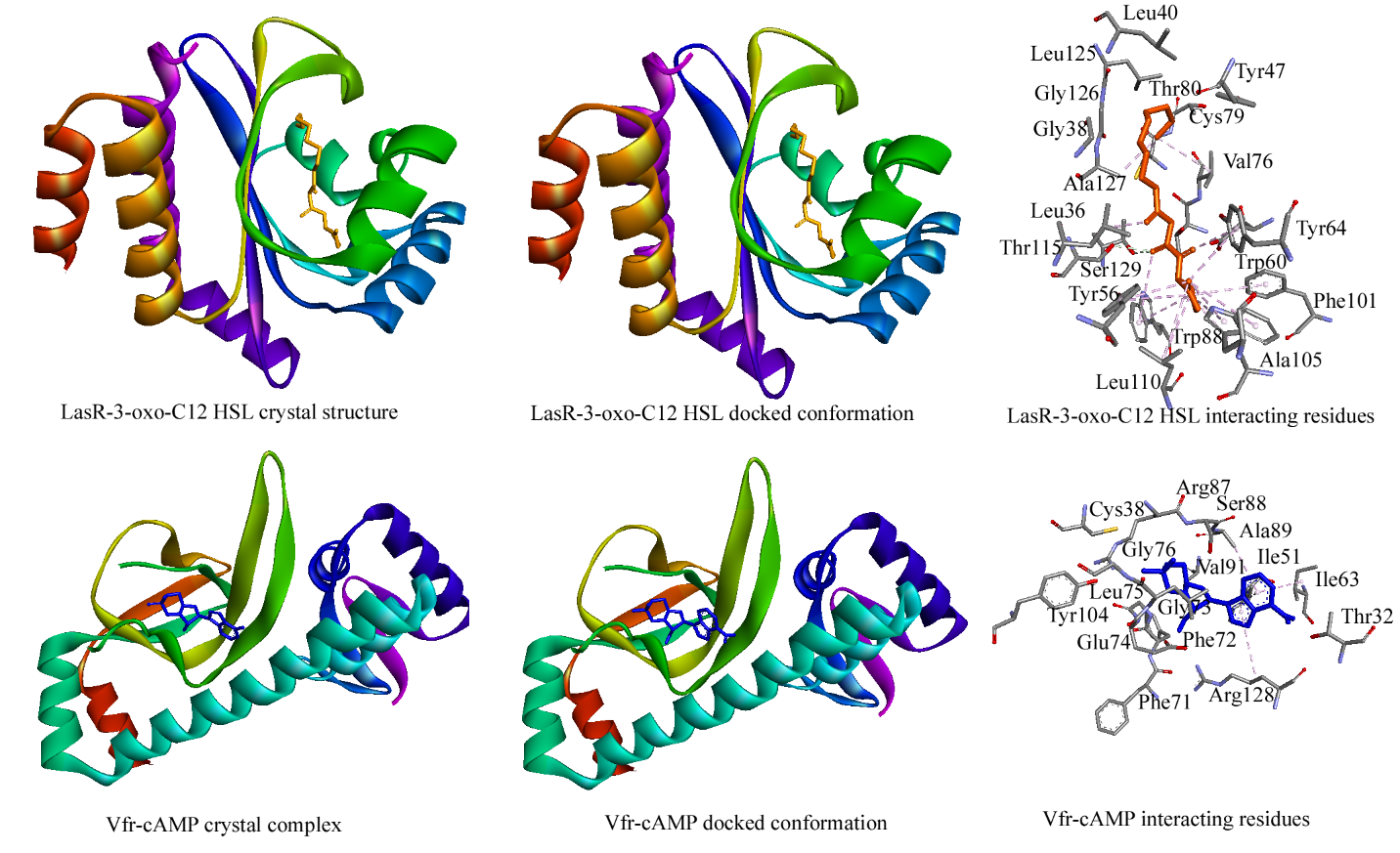
**

**Figure S5:** Comparison between crystal structures and docked conformations of ligand-protein complexes

**
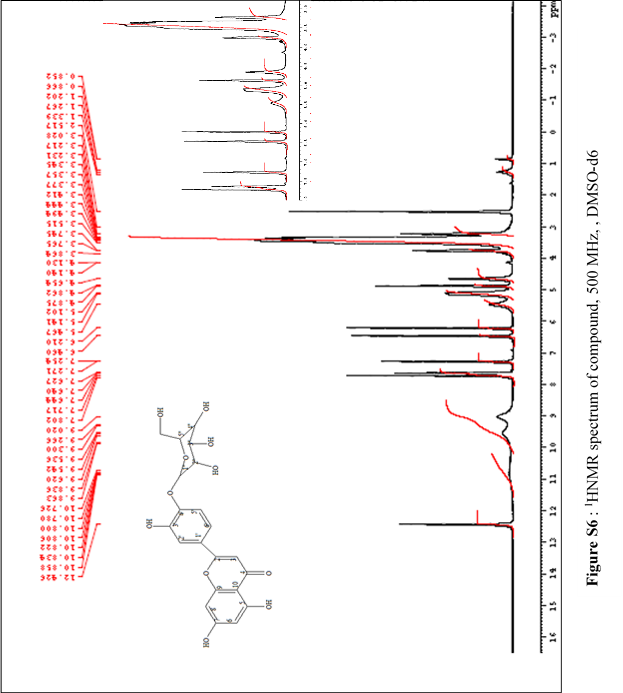
**

**
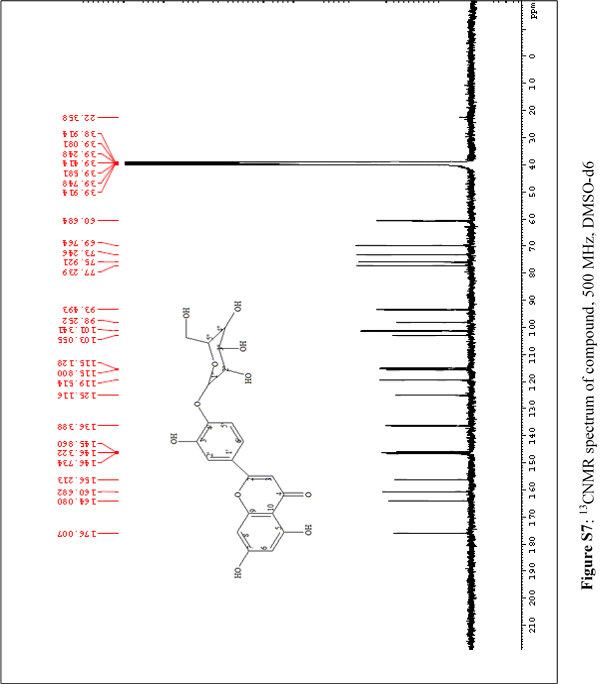
**


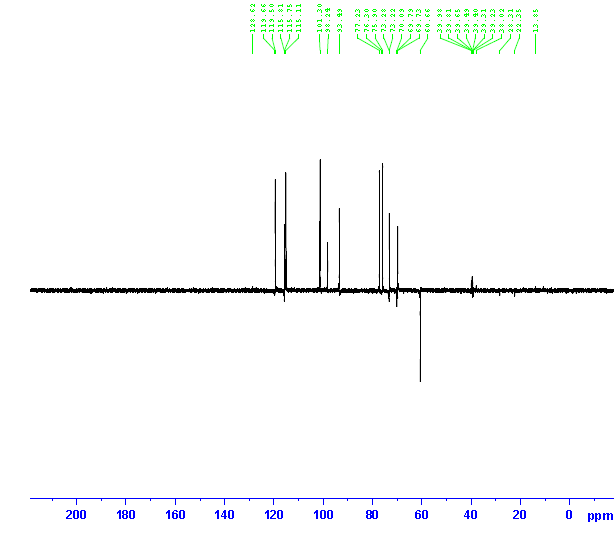


**Figure S8**: DEPT 135 spectrum of compound , DMSO-d6


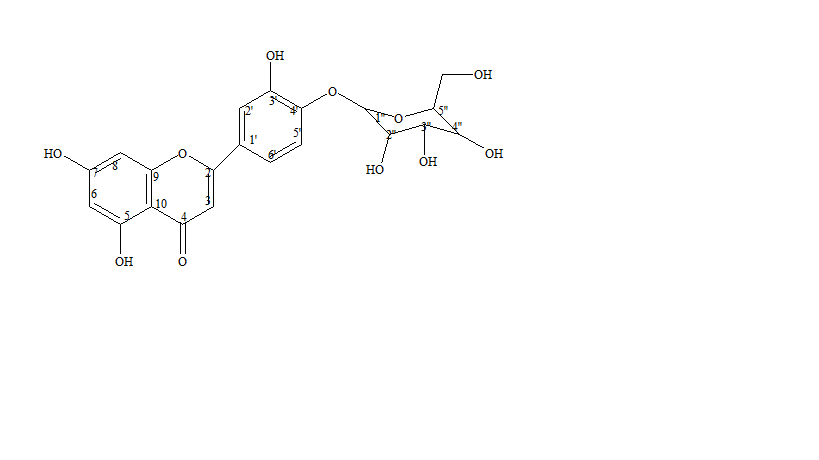


**Table S1:** ^1^H and ^13^C NMR data in ppm of compound QGP (in DMSO-*d_6_*):

|  | **δ_H_ *** | **δ_C_**** |
| --- | --- | --- |
| 2 | ----- | 146.3 |
| 3 | ---- | 136.4 |
| 4 | ----- | 176.0 |
| 5 | ----- | 160.7 |
| 6 | 6.21 1H (*br.s*) | 98.3 |
| 7 | ---- | 164.0 |
| 8 | 6.46 1H (*br.s*) | 93.5 |
| 9 | ---- | 156.2 |
| 10 | ---- | 103.0 |
| 1` | ---- | 125.1 |
| 2` | 7.71 1H (*d*, *J*= 1.5 Hz) | 115.1 |
| 3` | ---- | 145.9 |
| 4` | ---- | 146.7 |
| 5` | 7.26 1H (*d*,*J*=8.5 Hz) | 115.8 |
| 6` | 7.64 1H (*dd*, *J*=8.5 Hz, *J*=1.5 Hz) | 119.5 |
| 1`` | 4.86 (*d, J = 6.5* Hz) | 101.3 |
| 2`` | 3.21 1H (*m*) | 73.2 |
| 3`` | 3.35 1H (*m*) | 77.2 |
| 4`` | 3.41 1H (*m*) | 69.7 |
| 5`` | 3.51 1H (*m*) | 75.9 |
| 6`` | 3.74 1H (*m*), 3.23 1 H (*m*) | 60.7 |
| 5-OH | 12.43 IH (*s*) | ---- |
| 3-OH, 7-OH, 3`-OH | 10.80, 9.54, 9.02 1H (each *br.s*) | ---- |
| Glucose − OHs | 4.60 – 5.70 (broad peaks) | ---- |

*Assignment based on COSY experiments. ** Assignment based on DEPT-135 and HMQC experiments.

**
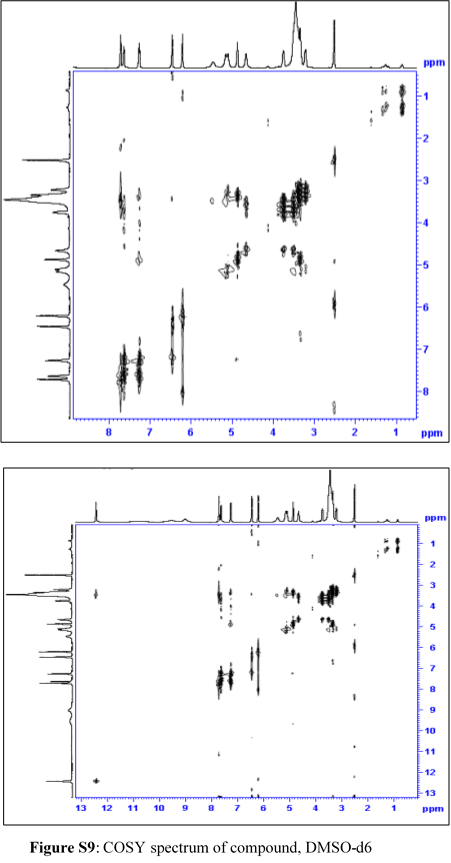
**

**
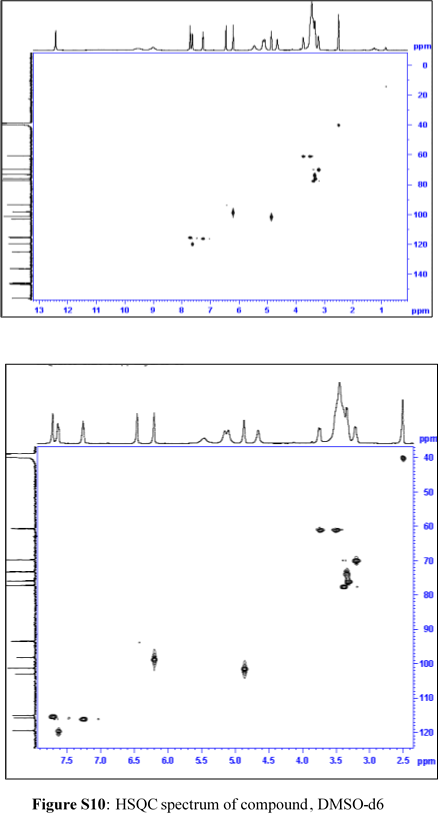
**

**
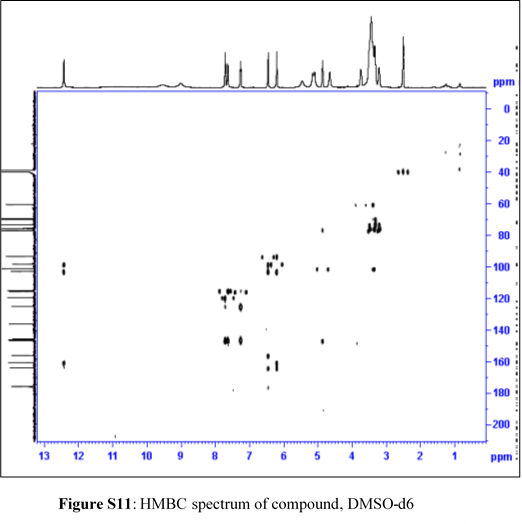
**

**Table S1:** ^1^HNMR* and ^13^C NMR* data in ppm (multiplicity, *J* in Hz) of the compound (in DMSO-d6):

| **No** | **δ_H_** | **δ_C_** |
| --- | --- | --- |
| 2 | ----- | 146.3 |
| 3 | ---- | 136.4 |
| 4 | ----- | 176.0 |
| 5 | ----- | 160.7 |
| 6 | 6.21 (1H, *s*) | 98.3 |
| 7 | ---- | 164.0 |
| 8 | 6.46 (1H, *s*) | 93.5 |
| 9 | ---- | 156.2 |
| 10 | ---- | 103.0 |
| 1* | ---- | 119.5 |
| 2* | 7.71 (1H, d, *J*= 1.5 Hz) | 115.1 |
| 3* | ---- | 146.7 |
| 4* | ---- | 145.9 |
| 5* | 7.26 ( 1H, d *J*=8.5 Hz) | 115.8 |
| 6* | 7.64 (1H, dd, J=8.5 Hz, *J*=1.5 Hz) | 119.5 |
| 1** | 4.86 (br., *s*) | 101.3 |
| 2** | 3.21 ( 1H, m) | 73.2 |
| 3** | 3.35 ( 1H, m) | 77.2 |
| 4** | 3.41 (1H, m) | 69.7 |
| 5** | 3.51 (1H, m) | 75.9 |
| **6**** | 3.74 ( 1H, m) | 60.7 |

* Assignment based on COSY experiments. ** Assignment based on DEPT 135, HMQC and HMBC experiments.

**Table S2:** Molecular interaction of QGP with Vfr and LasR

| **Type of interaction** | **Nature of force** | **Bond distance (Å)** | **Binding free energy, ΔG (kcal/mol)** | **Binding affinity, *K*_d_ (M^-1^)** |
| --- | --- | --- | --- | --- |
| **Vfr-QGP complex** | | | | |
| Unk:O5 - Val136  Unk:O10 - Arg128  Unk:O7 - Ile32  Unk:O7 - Arg87  Unk:O7 - Val91  Unk:O6 - Val136  Unk:O3 - Leu129  Unk - Ile51  Unk - Ala89  Unk - Val91  Unk - Ile51  Unk - Ala89 | Hydrophobic (alkyl)  Hydrophobic (alkyl)  Hydrophobic (alkyl)  Hydrophobic (alkyl)  Hydrophobic (alkyl)  Hydrophobic (alkyl)  Hydrophobic (alkyl)  Hydrophobic (π-alkyl)  Hydrophobic (π-alkyl)  Hydrophobic (π-alkyl)  Hydrophobic (π-alkyl)  Hydrophobic (π-alkyl) | 5.09  4.37  3.66  4.24  4.84  4.77  4.45  5.09  4.44  5.23  4.55  4.52 | -6.83 | 1.02 × 10^5^ |
| **Vfr-cAMP complex** | | | | |
| Unk - Ile51  Unk - Arg128  Unk - Ile51  Unk - Ile63  Unk - Ala89 | Hydrophobic (π-alkyl)  Hydrophobic (π-alkyl)  Hydrophobic (π-alkyl)  Hydrophobic (π-alkyl)  Hydrophobic (π-alkyl) | 4.55  5.44  5.49  5.03  4.89 | -8.91 | 3.43 × 10^6^ |
| **LasR-QGP complex** | | | | |
| Arg61:NH2 - Unk  Arg61:NE - Unk  Arg61 - Unk  Unk:O10 - Ile52  Unk:O11 - Ile52  Unk:O6 - Arg61  Unk - Ile52  Unk - Ile52  Unk - Arg61 | Electrostatic  Hydrogen bond  Hydrophobic (alkyl)  Hydrophobic (alkyl)  Hydrophobic (alkyl)  Hydrophobic (alkyl)  Hydrophobic (π-alkyl)  Hydrophobic (π-alkyl)  Hydrophobic (π-alkyl) | 3.72  4.14  5.07  3.72  5.14  3.28  5.08  4.72  5.31 | -5.98 | 2.43 × 10^4^ |
| **LasR-3-oxo-C12-HSL complex** | | | | |
| Unk:O9 - Thr115:OG1  Unk:O9 - Ser129:OG  Unk - Ala105  Unk - Leu110  Unk:C21 - Ala127  Unk:O12 - Leu36  Unk:C21 - Val76  Unk:C21 - Cys79  Unk:O6 - Leu110  Unk - Tyr56  Unk - Tyr56  Unk - Trp60  Unk - Trp60  Unk - Trp60  Unk - Tyr64  Unk - Tyr64  Unk - Trp88  Unk - Trp88  Unk - Phe101 | Hydrogen Bond  Hydrogen Bond  Hydrophobic (alkyl)  Hydrophobic (alkyl)  Hydrophobic (alkyl)  Hydrophobic (alkyl)  Hydrophobic (alkyl)  Hydrophobic (alkyl)  Hydrophobic (alkyl)  Hydrophobic (π-alkyl)  Hydrophobic (π-alkyl)  Hydrophobic (π-alkyl)  Hydrophobic (π-alkyl)  Hydrophobic (π-alkyl)  Hydrophobic (π-alkyl)  Hydrophobic (π-alkyl)  Hydrophobic (π-alkyl)  Hydrophobic (π-alkyl)  Hydrophobic (π-alkyl) | 3.38  2.76  4.78  4.49  3.85  3.53  4.34  3.62  4.78  5.49  4.33  5.32  3.93  4.89  4.03  4.37  5.18  4.99  5.15 | -9.10 | 4.72 × 10^6^ |
